# Evidence of the Impact of the *Tips From Former Smokers* Campaign: Results From the Behavioral Risk Factor Surveillance System

**DOI:** 10.5888/pcd16.190110

**Published:** 2019-10-10

**Authors:** Kevin C. Davis, Rebecca Murphy-Hoefer, Burton Levine, Brian A. King, Sean Hu, Robert Rodes

**Affiliations:** 1 RTI International, Center for Health Policy Science and Tobacco Research, Research Triangle Park, North Carolina; 2 Centers for Disease Control and Prevention, Office on Smoking and Health, Atlanta, Georgia

## Abstract

The *Tips From Former Smokers* campaign (Tips) has demonstrated significant impact as a population-based intervention for smoking cessation in the United States. Since 2012, evaluations of Tips have relied on web-panel data to attribute the campaign to smoking cessation outcomes. We re-examined the relationship between market-level doses of the campaign and quit attempts by using Behavioral Risk Factor Surveillance System (BRFSS) data to triangulate prior findings. We found that Tips was associated with increased quit attempts among smokers, which validates prior evaluation research on the impact of Tips. These results suggest that continued investments in Tips may help sustain its impact on cessation-related outcomes.

**Summary**

**What is already known on this topic?**

Research using national online surveys indicates that increases in market-level media buys for the *Tips From Former Smokers* campaign (Tips) television advertisements is associated with increased attempts to quit smoking among cigarette smokers.

**What is added by this report?**

This report validates findings of prior research and addresses potential limitations of research based on web surveys by demonstrating a dose–response relationship between Tips exposure and quit attempts among smokers in the Behavioral Risk Factor Surveillance System, a nationally representative surveillance system.

**What are the implications for public health practice?**

This study affirms that the more exposure to Tips that smokers receive, the more likely they are to make a quit attempt. These findings support year-round implementation of the campaign to maintain smokers’ motivation to quit and increase their likelihood of quitting for good.

## Objective

Since 2012, the Centers for Disease Control and Prevention’s (CDC’s) *Tips From Former Smokers *campaign (Tips) has been the largest national tobacco education mass media campaign focusing on smoking cessation among adult smokers. Several studies have used multiple methods to demonstrate the campaign’s impact on cessation behavior, including pre–post cohort designs (1,2), randomizations of campaign media buys (3), and cross-sectional time series analyses (4). Although these studies relied on probability-based web samples, results have not been replicable with other national surveillance systems. This triangulation is important given the public investment in the campaign and for informing future evaluation planning. In this study, we address this by re-examining the relationship between *Tips* exposure and quit attempts by using a large non-web survey source, the Behavioral Risk Factor Surveillance System (BRFSS). BRFSS is a national surveillance system that uses landline and mobile phone cross-sectional surveys among US adults. Using these data, we estimated the association between market-level doses of Tips media buys and quit attempts among adult cigarette smokers.

## Methods

We used BRFSS data collected from 2011 to 2015, corresponding to a pre-Tips baseline survey (2011) and serial cross-sectional surveys during the first 4 years of the campaign (2012–2015). BRFSS provides state-specific and national estimates among noninstitutionalized US adults. Surveys were conducted via telephone through random sampling of landline and cellular telephone sampling frames. Data were weighted to represent the US adult population. Tips gross ratings points (GRPs), a continuous measure of campaign dose that varies across US designated market areas (DMAs) and time (4), were merged with BRFSS data by respondents’ DMA of residence and survey completion date. GRPs are derived from Nielsen television ratings for programs during which Tips ads aired and were provided by the campaign’s media buyer. GRP categories represent equal-width ranges of observed GRPs in the analytic data from 2012 to 2015. Predicted values for incidence of past-year quit attempts are provided for each GRP category. We limited analyses to current adult cigarette smokers, defined as having smoked at least 100 cigarettes during their lifetime and currently smoking “some days” or “every day.”

The outcome variable was having made a quit attempt in the past 12 months. The primary independent variable was cumulative weekly Tips GRPs in the respondent’s DMA during the past year. Following methods used elsewhere (4–8), we used weighted logistic regression to estimate the odds of a past-year quit attempt as a function of cumulative past year Tips GRPs. To allow for nonlinearity in this relationship, we also estimated this model using the square root of cumulative GRPs to capture diminishing returns at higher GRPs (4). We calculated odds ratios (ORs) per unit increase of 5,000 market-level GRPs per year, an increment corresponding to CDC’s *Best Practices* *for Comprehensive Tobacco Control Programs *for dosing of tobacco education campaigns, which has been used as a dose increment in prior studies (6,9). Each model controlled for respondents’ sex, age, race/ethnicity, educational attainment, and state cigarette taxes. To account for time-invariant differences in quit attempts across states, we included separate indicators for each state. All models accounted for household clustering and survey sample design using SAS (SAS Institute, Inc) survey estimators. Lastly, we included a weekly time trend variable to account for underlying trends in cessation behavior not attributable to Tips.

## Results

The final analytic sample included 357,180 current smokers. The sample was 59.1% female; 14.8% were aged 18–34, 29.4% were aged 35–54, and more than half (55.8%) were aged 55 or older. Most of the sample (79.1%) was non-Hispanic white; the remaining sample was 8.2% non-Hispanic black, 6.7% Hispanic, and 6.0% non-Hispanic other race. An estimated 60.2% of smokers in the sample indicated making a quit attempt in the past year. For every 5,000 GRP increase in cumulative exposure to Tips ads annually, odds of reporting a past-year quit attempt were significantly increased (OR = 1.16; 95% confidence interval [CI], 1.06–1.27; *P* = .01) (Table 1). Past-year GRPs measured in curvilinear form yielded similar results, showing that increased past-year GRPs were associated with increased odds of a past-year quit attempt (OR = 1.11; 95% CI, 1.05–1.17; *P* = .001). Using predicted values from the curvilinear model (Table 1), we estimated that 54.8% of respondents who received no GRPs made a quit attempt in the past year compared with a 56.1% quit attempt rate among those who received between 2,160 and 3,240 GRPs and a 59.3% quit attempt rate among those who received 3,241 to 4,316 GRPs (Table 2).

**Table 1:** Association Between Cumulative Past-Year GRPs and Past-Year Incidence of Making a Quit Attempt Among Current Cigarette Smokers^a^ (N = 357,180), *Tips From Former Smokers* Campaign, 2011–2015

Independent Variable^b^	Linear Model		Nonlinear Model	
	Odds Ratio (95% Confidence Interval)	*P* Value	Odds Ratio (95% Confidence Interval)	*P* Value
Annual GRPs (in 5,000s)	1.16 (1.06–1.27)	.01	1.11 (1.05–1.17)	.001
**Sex**				
Male	1 [Reference]			
Female	1.15 (1.12–1.18)	<.001	1.15 (1.12–1.18)	<.001
**Age, y**				
18–24	2.08 (1.97–2.20)	<.001	2.08 (1.97–2.20)	<.001
25–34	1.69 (1.61–1.77)	<.001	1.69 (1.61–1.77)	<.001
35–44	1.44 (1.37–1.50)	.03	1.44 (1.37–1.50)	.03
45–54	1.21 (1.16–1.26)	<.001	1.21 (1.16–1.26)	<.001
55–64	1.18 (1.13–1.23)	<.001	1.18 (1.13–1.23)	<.001
≥65	1 [Reference]			
**Race/ethnicity**				
Non-Hispanic white	1 [Reference]			
Non-Hispanic African American	1.81 (1.72–1.89)	<.001	1.81 (1.72–1.89)	<.001
Hispanic	1.40 (1.32–1.48)	.04	1.40 (1.32–1.48)	.04
Non-Hispanic other race	1.24 (1.17–1.32)	<.001	1.24 (1.17–1.32)	.004
**Education**				
Less than high school	1.04 (0.99–1.09)	.42	1.04 (0.99–1.09)	.42
High school	0.97 (0.94–1.01)	<.001	0.97 (0.94–1.01)	<.001
Some college	1.09 (1.05–1.14)	<.001	1.09 (1.05–1.14)	<.001
Bachelor’s degree	1 [Reference]			
**State cigarette tax**	1.04 (0.97–1.13)	.27	1.04 (0.96–1.12)	.34

Abbreviation: GRPs, gross ratings points.

a Defined as having smoked at least 100 cigarettes during their lifetime and currently smoking “some days” or “every day.”

b Results determined by weight logistic regression. Each model controlled for weekly time trend and state fixed effects.

**Table 2:** Predicted Incidence of Making a Quit Attempt in the Past Year Among Current Cigarette Smokers^a^, by Cumulative Past-Year GRPs^b^, *Tips From Former Smokers* Campaign, 2011–2015

Past-Year GRPs	No.	Average Incidence of Past-Year Quit Attempt, %
0	92,773	54.8
0–1,080	150,029	55.4
1,081–2,160	97,060	56.7
2,161–3,240	16,256	56.1
3,241–4,316	1,062	59.3

Abbreviation: GRPs, gross ratings points.

a Defined as having smoked at least 100 cigarettes during their lifetime and currently smoking “some days” or “every day.”

b GRPs are derived from Nielsen television ratings for programs during which Tips ads aired and were provided by the campaign’s media buyer. GRP categories represent equal-width ranges of observed GRPs in the analytic data from 2012 to 2015.

## Discussion

BRFSS data showed that exposure to Tips is associated with higher annual quit attempt rates. This finding is consistent with evidence of the campaign’s impact on cessation behavior, which has focused primarily on its effects on more recent quitting behavior in the past 3 months (1–4). In general, these results are in accordance with the collective volume of media evaluation research that to date has predominantly documented positive effects from tobacco education campaigns (10). Therefore, our results indicate that continued investments in the Tips campaign can help maintain its cumulative impact on cessation-related outcomes.

The primary limitation of this study was that it relied on self-reported recall of potentially distant-past behavior (quit attempts during the past year), which may limit the validity of measurement of our outcome variable. In addition, the timing of Tips GRP delivery during the past year may not have corresponded with the specific timing of quit attempts made for some individuals. However, research shows that Tips GRPs are associated with intentions to quit smoking (4), and there are strong associations between weekly and with weekly calls to quitlines (11,12), which suggests reasonable alignment between the timing of GRP delivery and campaign-targeted outcomes.

Our results verify previous Tips campaign evaluation research, which relied on probability-based online surveys. This consistency of findings across multiple data sources and analytic methods further reinforces that the Tips campaign is an effective public health intervention for encouraging smokers to quit. Moreover, this study provides a practical example of using a national surveillance system with campaign exposure data for evaluation applications.

In summary, our results provide evidence of the positive impact of Tips on cessation behavior among smokers and validate previous evaluation research. More broadly, these results affirm that the more exposure smokers receive from the campaign, the more likely they are to make a quit attempt. Taken together, these findings support implementation of the campaign at adequate exposure levels consistent with CDC best practice recommendations (9) to maintain smokers’ motivation to quit and to increase their likelihood of quitting for good.
